# A role for the unfolded protein response stress sensor ERN1 in regulating the response to MEK inhibitors in KRAS mutant colon cancers

**DOI:** 10.1186/s13073-018-0600-z

**Published:** 2018-11-27

**Authors:** Tonći Šuštić, Sake van Wageningen, Evert Bosdriesz, Robert J. D. Reid, John Dittmar, Cor Lieftink, Roderick L. Beijersbergen, Lodewyk F. A. Wessels, Rodney Rothstein, René Bernards

**Affiliations:** 1 Division of Molecular Carcinogenesis, Oncode Institute, The Netherlands Cancer Institute, Plesmanlaan 121, Amsterdam, 1066 CX The Netherlands; 2 Department Genetics and Development, Columbia University Vagelos College of Physicians & Surgeons, New York, NY 10032 USA

**Keywords:** Ire1, ERN1, MEK inhibitor, Colon cancer, JNK, JUN

## Abstract

**Background:**

Mutations in *KRAS* are frequent in human cancer, yet effective targeted therapeutics for these cancers are still lacking. Attempts to drug the MEK kinases downstream of KRAS have had limited success in clinical trials. Understanding the specific genomic vulnerabilities of *KRAS*-driven cancers may uncover novel patient-tailored treatment options.

**Methods:**

We first searched for synthetic lethal (SL) genetic interactions with mutant *RAS* in yeast with the ultimate aim to identify novel cancer-specific targets for therapy. Our method used selective ploidy ablation, which enables replication of cancer-specific gene expression changes in the yeast gene disruption library. Second, we used a genome-wide CRISPR/Cas9-based genetic screen in *KRAS* mutant human colon cancer cells to understand the mechanistic connection between the synthetic lethal interaction discovered in yeast and downstream RAS signaling in human cells.

**Results:**

We identify loss of the endoplasmic reticulum (ER) stress sensor *IRE1* as synthetic lethal with activated *RAS* mutants in yeast. In *KRAS* mutant colorectal cancer cell lines, genetic ablation of the human ortholog of *IRE1*, *ERN1*, does not affect growth but sensitizes to MEK inhibition. However, an ERN1 kinase inhibitor failed to show synergy with MEK inhibition, suggesting that a non-kinase function of ERN1 confers MEK inhibitor resistance. To investigate how ERN1 modulates MEK inhibitor responses, we performed genetic screens in *ERN1* knockout *KRAS* mutant colon cancer cells to identify genes whose inactivation confers resistance to MEK inhibition. This genetic screen identified multiple negative regulators of JUN N-terminal kinase (JNK) /JUN signaling. Consistently, compounds targeting JNK/MAPK8 or TAK1/MAP3K7, which relay signals from ERN1 to JUN, display synergy with MEK inhibition.

**Conclusions:**

We identify the ERN1-JNK-JUN pathway as a novel regulator of MEK inhibitor response in *KRAS* mutant colon cancer. The notion that multiple signaling pathways can activate JUN may explain why *KRAS* mutant tumor cells are traditionally seen as highly refractory to MEK inhibitor therapy. Our findings emphasize the need for the development of new therapeutics targeting JUN activating kinases, TAK1 and JNK, to sensitize *KRAS* mutant cancer cells to MEK inhibitors.

**Electronic supplementary material:**

The online version of this article (10.1186/s13073-018-0600-z) contains supplementary material, which is available to authorized users.

## Background

Mutation of specific codons in one of the three *RAS* genes *HRAS*, *KRAS*, or *NRAS* converts these genes into oncogenes. These mutations are found in a wide variety of tumors, with very high incidences (> 50%) in pancreas and colon cancers [1]. Despite decades of research, generation of selective inhibitors of mutant RAS has proven to be difficult. Recently, allosteric inhibitors of KRAS G12C have been developed [2, 3], but the clinical effectiveness of these compounds remains to be established.

*RAS* genes are highly conserved in evolution. The yeast *Saccharomyces cerevisiae* has two *RAS* genes: *RAS1* and *RAS2*. These two genes are individually not required for cell viability. However, the double deletion mutant is inviable, indicating that the genes share an essential function [4]. A yeast *ras1Δ ras2Δ* deletion mutant can be rescued by ectopic expression of a human *RAS* gene [5]. Vice versa, mutating codon 19 into a valine converts yeast RAS into a constitutively active protein and this mutant yeast RAS can induce malignant transformation of mouse fibroblasts [6].

We searched for synthetic lethal (SL) genetic interactions with mutant *RAS* in yeast to identify novel cancer-specific targets for therapy. Our method uses selective ploidy ablation (SPA) and allows us to mimic cancer-specific gene expression changes in each of the 4800 nonessential deletion mutant strains in the yeast gene disruption library [7]. Using this approach, we found that inhibition of yeast unfolded protein response (UPR) genes is synthetic lethal with mutant *RAS*.

The UPR in yeast is mediated by Ire1 and Hac1 [8]. Ire1 is an endonuclease that upon endoplasmic reticulum (ER) stress splices *HAC1* mRNA. Hac1 is a transcription factor that executes the UPR by activating genes involved in ER homeostasis. The UPR, and the mechanism of activation by splicing of a specific mRNA, is conserved from yeast to humans. Mammalian cells have an *IRE1* ortholog named *ERN1*. Likewise, *HAC1* has a functional human homolog, *XBP1* [9]. In mammalian *KRAS* mutant colon cancer, we find that inhibition of MEK kinases is synthetic lethal with inhibition of the UPR. Our findings establish an unexpected link between MEK kinase signaling and the UPR executor ERN1 in human cancer.

## Methods

### Yeast screen

Wild-type RAS alleles were cloned into pWJ1512 using the A and B adaptamers [10]. Primers to obtain mutant RAS alleles (mutant sequence underlined) were RAS1(V19)-pWJ1512-F 5′ gaattccagctgaccaccATGCAGGGAAATAAATCAACTATAAGAGAGTATAAGATAGTAGTTGTCGGTGGAGTAGGCGTTGGTAAATCTGCTTTAAC, RAS2(V19)-pWJ1512-F 5′ gaattccagctgaccaccATGCCTTTGAACAAGTCGAACATAAGAGAGTACAAGCTAGTCGTCGTTGGTGGTGTTGGTGTTGGTAAATCTGCTTTG, pWJ1512-R 5′ gatccccgggaattgccatg.

The SPA protocol [7] was used to transfer plasmids into the arrayed gene disruption library [11]. Briefly, SPA is a yeast mating-based protocol that allows transfer of a plasmid from a special donor strain into a recipient strain followed by destabilization and counter-selection of the donor yeast chromosomes. The method was adapted for the RAS screen by adding 2% raffinose in addition to 2% galactose as a carbon source for the last two selection steps. In addition, selection steps for RAS2(V19) cells were 1 day longer because overall growth is slower in these strains.

### Cell culture, transfection and lentiviral infection

HEK293 cells were cultured in DMEM. All other cell lines were maintained in RPMI1640 medium containing 10% FBS and 1% penicillin/streptomycin at 37 °C and 5% CO_2_. All cell lines were purchased from the American Type Culture Collection (ATCC), STR profiled (by Eurofins Medigenomix Forensik GmbH, Ebersberg, Germany), and routinely tested negative for mycoplasma.

Transfection of HEK293 cells with linear polyethylenimine (PEI) 25K from Polysciences (cat# 23966-2) and subsequent infection of target cells was done as described previously [12]. For knockout of individual genes, the following single-guide (sg) RNAs were cloned in the lentiCRISPR version 2.1 (LC2.1) vector by Gibson cloning: sgERN1-A, 5′-ACATCCCGAGACACGGTGGT-3′; sgERN1-B, 5′-GATGGCAGCCTGTATACGCT-3′; sgDET1, 5′-ACGTGCAGCAGTGTCGCATA-3′; sgCOP1, 5′-AAGCTCCTTCTCCATCACAC-3′. Non-targeting (NT) sgRNA 5′-ACGGAGGCTAAGCGTCGCAA-3′ was used as a control.

### Cell proliferation assays and growth curves

For long-term cell proliferation assays, cells were seeded in six-well plates at densities ranging from 1 to 2 × 10^4^ cells per well and cultured with or without inhibitors, as indicated. When control cells reached confluency, all cells were fixed in 4% formaldehyde and stained with 0.1% crystal violet (in water).

Live cell growth was measured by automated determination of confluency every 4 h using IncuCyte Zoom (Essen Bioscience). Between 600 and 800 cells were plated per well of a 96-well plate, and experiments were carried out in triplicates.

MEK inhibitors selumetinib (AZD6244) and trametinib (GSK1120212) were purchased from Selleck Chemicals and kept as 10 mM stock solutions in DMSO. ERN1 inhibitor (compound 18) and JNK inhibitor SR-3306 were kindly provided by Astex Pharmaceuticals. TAK1 inhibitor was purchased from Merck as (5Z)-7-Oxozeaenol (CAS 66018-38-0).

### Protein lysate preparation and western blot analysis

Cells were lysed and western blots performed as described previously [12]. Primary antibodies against HSP90 (sc-13119), p-JUN (sc-822), and ERK2 (sc-154) were purchased from Santa Cruz. Antibodies against ERN1 (#3294), GAPDH (#5174), p-ERK1/2 (#9101), and JUN (#9165) were from Cell Signaling. Antibodies against COP1 (Genentech, 28A4) and DET1 (Genentech, 3G5) were a gift from Vishva Dixit, Genentech. Secondary antibodies were obtained from Bio-Rad Laboratories.

### Total RNA isolation and quantitative RT-PCR

Total RNA was isolated and purified using Quick-RNA™ MiniPrep (Zymo Research), and reverse transcription was performed with Maxima Universal First Strand cDNA Synthesis Kit (Thermo Fisher Scientific, #K1661).

The 7500 Fast Real-Time PCR System from Applied Biosystems was used to measure mRNA levels, which were normalized to the expression of GAPDH, in triplicates. The following primer sequences were used in the SYBR® Green master mix (Roche): *GAPDH*-Fw, AAGGTGAAGGTCGGAGTCAA; *GAPDH*-Rev, AATGAAGGGGTCATTGATGG; *ERN1*-Fw, AGCAAGCTGACGCCCACTCTG; *ERN1*-Rev, TGGGGCCCTTCCAGCAAAGGA; CD59-Fw, CAGTGCTACAACTGTCCTAACC; CD59-Rev, TGAGACACGCATCAAAATCAGAT; JUN-Fw, AACAGGTGGCACAGCTTAAAC; JUN-Rev, CAACTGCTGCGTTAGCATGAG; JNK1-Fw, TGTGTGGAATCAAGCACCTTC; JNK1-Rev, AGGCGTCATCATAAAACTCGTTC; JNK2-Fw, GAAACTAAGCCGTCCTTTTCAGA; JNK2-Rev, TCCAGCTCCATGTGAATAACCT. To detect human XBP1 mRNA, we used hXBP1-Fw, GAAGCCAAGGGGAATGAAGT and hXBP1-Rev, GCTGGCAGGCTCTGGGGAAG. To detect human spliced Xbp1, hXBP1-Rev was used with hXBP1spl-Fw, TGCTGAGTCCGCAGCAGGTG, as designed previously [13].

### CRISPR-Cas9 resistance screen

To generate *ERN1* knockout cells that would not contain the same tracer sequence as currently available CRISPR libraries and thus be suitable for subsequent genome-wide screening, we used a dual vector doxycycline-inducible CRISPR/Cas9 system made on the basis of FH1tUTG [14], as previously described [15]. Single-cell clones were tested for *ERN1* knockout by western blot and by measuring the levels of spliced XBP1 using quantitative RT-PCR as described above.

Version 2 of the human genome-scale CRISPR-Cas9 knockout (GeCKO) half-library A—consisting of 65,383 sgRNAs in lentiviral vectors [16] (Addgene #1000000048)—was used to infect LoVo *ERN1* knockout cells with a transduction efficiency of 20% in a sufficient cell number to achieve a 180-fold library coverage. After 48 h, cells were replated and viral supernatant was replaced by medium containing puromycin (2 μg/ml) to select for infected cells for 2 days. After additional 4 days of growth, cells were harvested, a T_0_ sample was taken, and the rest of the cells were reseeded and cultured in the presence or absence of MEK inhibitors selumetinib and trametinib, in two biological replicates each, for 4 weeks. Genome-integrated sgRNA sequences were PCR amplified and their respective abundance was determined as described previously [17]. The abundance of each sgRNA in the treated versus untreated pools was determined by massively parallel sequencing on an Illumina HiSeq 2500 platform. Statistical analysis was performed using DESeq version 1.24.0. The hit selection was based on the overlap between selumetinib and trametinib screens for the genes for which at least one of the sgRNAs meet the following criteria: (A) log_2_ fold change (of treated over untreated samples) ≥ 7, (B) baseMeanA (mean number of reads in the untreated sample) ≥ 50, and (C) adjusted *p* value ≤ 0.1. The results overview of the CRISPR screen can be found in Additional file 1: Tables S5 and S6.

## Results

### RAS synthetic lethality screens in yeast

To discover genetic interactions with mutant yeast *RAS*, we expressed the constitutively active *RAS* alleles, *RAS1(V19)*, and *RAS2(V19)*, in the collection of ~ 4800 yeast strains in which each individual nonessential gene is deleted [11]. To discriminate between effects due to ectopic expression of the *RAS* alleles and those due to the specific *RAS* gene mutations, we also screened the wild-type *RAS1(wt)* and *RAS2(wt)* alleles. Additionally, we screened cells harboring an empty vector as a control. Median-normalized growth values were used to calculate the growth ratios between experimental and vector control colonies (Additional file 2: Figure S1A) [18]. We have shown previously that a screen organizes related genes based on phenotype, and these genes exhibit a high density of interactions within the group. The CLIK algorithm plots this density of interactions from the ranked screen results to determine a cutoff for the screen [19]. CLIK analysis of the *RAS1(V19)* and *RAS2(V19)* screens yielded respectively 151 and 450 strains with a growth defect, which corresponds to a twofold difference in growth compared to the population median in both screens (Additional file 1: Table S1). Although no CLIK groups were identified for the *RAS1(wt)* and *RAS2(wt)* screens, the same twofold growth difference cutoff was applied, which yields 14 affected strains from each screen (Additional file 2: Figure S1B-E) indicating that the majority of SL interactions are specific to the activated *RAS* mutants. Interestingly, most SLs from the *RAS1(V19)* were also found in the *RAS2(V19)* screen (Additional file 2: Figure S1F). The growth effects in the *RAS1(V19)* and *RAS2(V19)* screens were highly correlated although the effect was more severe in the *RAS2(V19)* screen (Additional file 2: Figure S1G). This finding suggests that the yeast *RAS* genes form a quantitative redundant pair [20].

**Fig. 1:**
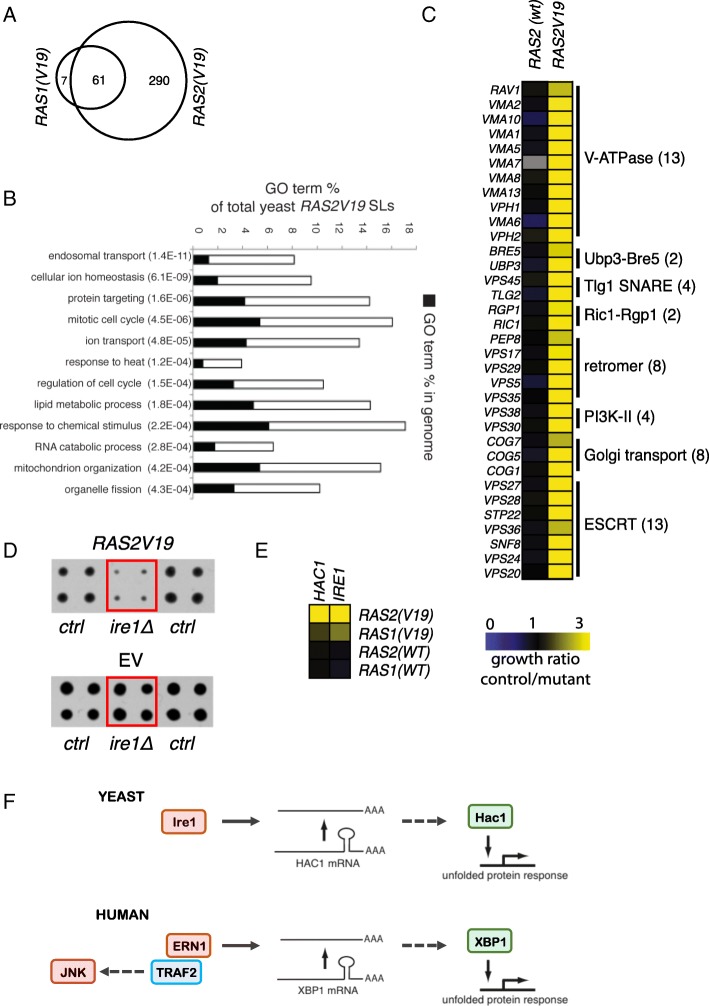
Unfolded protein response (UPR) executors are synthetic lethal with mutant RAS in *S. cerevisiae*. **a** Venn diagram showing the overlap of the *RAS* synthetic lethal (SL) gene deletion strains identified in the *RAS1(V19)* and *RAS2(V19)* genetic screens. **b** Gene Ontology (GO) enrichment analysis on the SL gene deletion strains from the *RAS2(V19)* screen identifies a variety of biological processes, including endosomal transport and protein targeting. **c** List of genes coding for protein complexes among the validated list of *RAS2(V19)* SL gene deletion mutants. Higher values correspond to stronger growth arrest in the presence of mutant *RAS*. The pathways and complexes in which the genes are involved are indicated. **d** The effect of the deletion of the UPR stress sensor *IRE1* (*ire1Δ*) in *RAS2(V19)* screen (top) and in the empty vector (EV) control background (bottom). **e** Control vs mutant growth ratios of the UPR genes *IRE1* and *HAC1*. Higher values correspond to stronger growth arrest in the presence of mutant *RAS*. **f** Schematic representation of the evolutionary conserved mechanism of UPR execution in yeast (top) and humans (bottom). Ire1 is responsible for the editing of *HAC1* mRNA which produces an active executor of the UPR. *ERN1* is the human ortholog of yeast *IRE1*; *XBP1* is a functional human homolog of *HAC1*

To validate the deletion mutants from the SL screens, the strains that showed an SL interaction were rescreened with a mutant or wild-type *RAS* allele. Forty-six percent of the *RAS1(V19)* and 79% of the *RAS2(V19)* SLs from the primary screen also had a growth defect (> 2 times smaller than control) in the validation screen (Additional file 1: Tables S2-S5). Ninety percent of the validated hits of the *RAS1(V19)* screen overlapped with the *RAS2(V19)* screen. The gene deletions from the *RAS1(wt)* and *RAS2(wt)* screens did not validate in a second screen, indicating that the SLs are specific to the mutant alleles and that *RAS1(V19)* interacts with a subset of the *RAS2(V19)* SLs (Fig. 1a). We decided to focus on the genes from the *RAS2* mutant screen due to the higher number of interactions and the higher validation rate. In addition, almost all of the *RAS1* mutant gene deletions were also found and validated in the *RAS2* screen.

An encouraging sign of the validity of our screen was the recapitulation of the synthetic lethal interaction between *RAS2(V19)* and *SIN4*. *SIN4* is a component of the mediator transcription complex *(MED16)*, and its interaction with *RAS2(V19)* has been described before [21]. Additionally, we found that another mediator component, *PGD1* (*MED3*), is synthetic lethal with *RAS2(V19)*.

We performed a Gene Ontology (GO) enrichment analysis on the SLs from the *RAS2(V19)* screen, which identified a variety of biological processes enriched in this screen, including endosomal transport and protein targeting (Fig. 1b). This finding indicates that cells expressing *RAS2(V19)* are highly dependent on intracellular protein transport. We further analyzed the validated list of *RAS2(V19)* SLs by identifying protein complexes from which two or more members were present, based on Benschop et al [22]. Again, in this analysis, we recovered several complexes involved in endosomal transport (Fig. 1c). Based on the dependence of cells expressing *RAS2(V19)* on intracellular transport, we hypothesized that ER homeostasis was disturbed in these cells, which would be consistent with the work of Leber et al [23]. To test this hypothesis, we compared our list of *RAS2(V19)* SLs to lists of strains that are sensitive to ER stress agents [24]. We confirmed a significant overlap with strains sensitive to β-mercaptoethanol, DTT, and tunicamycin (*P* = 3.07E−05, hypergeometric test; Additional file 1: Table S6), suggesting that ER homeostasis is disturbed by *RAS2(V19)*.

Mutant RAS is known to inhibit the production of GPI-anchors at the ER [25]. This inhibition likely contributes to permanent ER stress in cells expressing *RAS2(V19)*. To test this theory, we compared the effect of expressing *RAS2(V19)* with directly inhibiting GPI-anchor production by analyzing the synthetic lethal genetic interactions of *ERI1*, a non-essential component of the GPI-GnT enzyme [26]. Again, we found a significant overlap between the *RAS2(V19)* SLs lists and the list of *ERI1* genetic interactions (*P* = 8.60E−09, hypergeometric test; Additional file 1: Table S7). The strongest negative genetic interaction of *ERI1* is with *IRE1*, an important regulator of the UPR. Additionally, *ERI1* shows a strong negative genetic interaction with *HAC1*, a downstream target of Ire1. The UPR is a signaling route that restores ER homeostasis and *ire1Δ* and *hac1Δ* strains are highly sensitive to ER stress agents (Additional file 1: Table S6). Importantly, we found both *IRE1* and *HAC1* are *RAS2(V19)* SLs (Fig. 1d, e), indicating that ER homeostasis is disturbed in *RAS2(V19)* expressing cells and that these cells are dependent on the UPR.

### Genetic ablation of ERN1 in KRAS mutant colon cancer cells

**Fig. 2:**
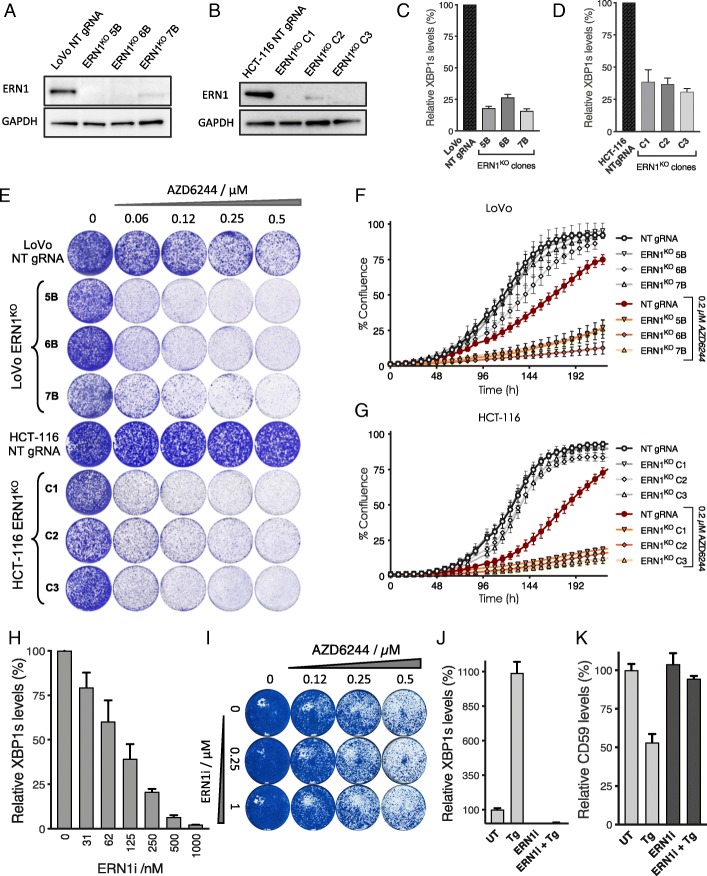
Effects of ERN1 inhibition in *KRAS* mutant human colon cancers. **a**, **b** Western blot analysis of *ERN1* expression in control cells expressing non-targeting (NT) gRNA and LoVo *ERN1*^KO^ clones 5B, 6B, and 7B (**a**) and HCT-116 *ERN1*^KO^ clones C1, C2, and C3 (**b**). **c**, **d** qPCR analysis of spliced XBP1 mRNA (XBP1s) in control cells expressing non-targeting (NT) gRNA and LoVo ERN1^KO^ clones 5B, 6B, and 7B (**c**) and HCT-116 ERN1^KO^ clones C1, C2, and C3 (**d**). Error bars indicate standard deviation calculated from three biological replicates. **e** Representative colony formation assays of three different *ERN1*^KO^ clones compared to the non-targeting (NT) gRNA expressing control cells in the *KRAS* mutant LoVo (top) and HCT-116 colon cancer cells (bottom). Cells were maintained in the indicated range of concentrations of the MEK inhibitor selumetinib (AZD6244) for 10 days, stained and photographed. **f**, **g** Live cell proliferation assay (IncuCyte®) of control (NT gRNA) and *ERN1*^KO^ cells following exposure to the MEK inhibitor AZD6244. Error bars indicate standard deviation of three replicate experiments. **h** qPCR analysis of spliced XBP1 mRNA (XBP1s) levels following exposure of LoVo cells to increasing concentrations of the ERN1 kinase inhibitor. Error bars indicate standard deviation calculated from three replicate experiments. **i** Colony formation assay showing the effect of ERN1 kinase inhibitor on the proliferation of *KRAS* mutant LoVo cells in the presence of the indicated concentrations of the MEK inhibitor AZD6244. **j** Quantification of spliced XBP1 mRNA (XBP1s) levels following 1 h treatment with 100 nM of ER stress inducer thapsigargin (Tg) in the presence and absence of the ERN1 kinase inhibitor. **k** Quantification of the mRNA levels of the RIDD target CD59 after 1 h treatment with 100 nM thapsigargin (Tg) in the presence and absence of the ERN1 kinase inhibitor

The UPR, and the mechanism of activation by splicing of a specific mRNA, is conserved from yeast to humans (Fig. 1f). Mammalian cells have an *IRE1* ortholog, named *ERN1*, while *HAC1* has a functional human homolog named *XBP1*, whose mRNA is spliced by the ERN1 endonuclease domain to form the active, protein-coding XBP1 spliced (XBP1s) form [9]. To test whether *ERN1* is essential in cells with active RAS signaling, we created *ERN1* knockout (KO) LoVo, HCT-116, SW480, and DLD1 *KRAS* mutant colon cancer cells using lentiviral CRISPR-Cas9 vectors. *ERN1* ^KO^ cells had an absence of ERN1 protein and a strong decrease in spliced XBP1 (XBP1s) (Fig. 2a–d). We found that the proliferation of *ERN1*^KO^ cells was similar to control cells expressing non-targeting (NT) gRNA, indicating that the synthetic lethal interaction between RAS and the UPR is not conserved between yeast and human cells. However, since yeast cells are missing the RAF/MEK/ERK MAPK cascade, we investigated the proliferation of the *KRAS* mutant *ERN1*^KO^ cells in the presence of the MEK inhibitor selumetinib (AZD6244). Interestingly, we found increased MEK inhibitor sensitivity in all *ERN1*^KO^ LoVo, HCT-116, and SW480 cell clones, both in short-term and in long-term assays (Fig. 2e–g and Additional file 2: Figure S2A-C). In DLD1 cells, no effect on selumetinib response was observed upon *ERN1* KO (Additional file 2: Figure S2D-F). These data indicate that a subset of *KRAS* mutant colon cancer cells can be sensitized to MEK inhibition by loss of ERN1.

### Pharmacologic inhibition of ERN1

The ERN1 protein contains both an endonuclease and a kinase domain. A specific inhibitor of ERN1 kinase activity has been developed that results in allosteric inhibition of the endonuclease activity, referred to as compound 18 by Harrington et al [27]. We tested the potency of this inhibitor in LoVo cells by measuring XBP1s levels 24 h after treatment with increasing amounts of ERN1 inhibitor. The compound proved effective with an IC50 of approximately 100 nM (Fig. 2h). Next, we tested whether treatment with this potent ERN1 inhibitor would increase the sensitivity of LoVo cells to the MEK inhibitor. To our surprise, inhibition of ERN1 endonuclease activity was not sufficient to recapitulate the phenotype of the genetic ablation of *ERN1* (Fig. 2i).

ERN1 is able to cleave other mRNAs besides XBP1, a process termed regulated IRE1-dependent decay (RIDD) [28]. We tested whether the ERN1 inhibitor interfered with RIDD by stressing LoVo cells with the ER stress-inducing agent thapsigargin (Tg) both in the absence and presence of the ERN1 kinase inhibitor. One of the RIDD targets is *CD59* [29]. As expected, XBP1 levels increased and CD59 mRNA levels decreased upon treatment with Tg. In the presence of the ERN1 inhibitor, XBP1 splicing was not increased and *CD59* mRNA levels did not decrease upon treatment with Tg (Fig. 2j, k). These data show that RIDD is effectively inhibited by the ERN1 inhibitor and that RIDD targets are unlikely to be involved in the sensitization of *ERN1*^KO^ cells to the MEK inhibitor.

### Genome-wide screen reveals ERN1-JNK-JUN signaling axis

**Fig. 3:**
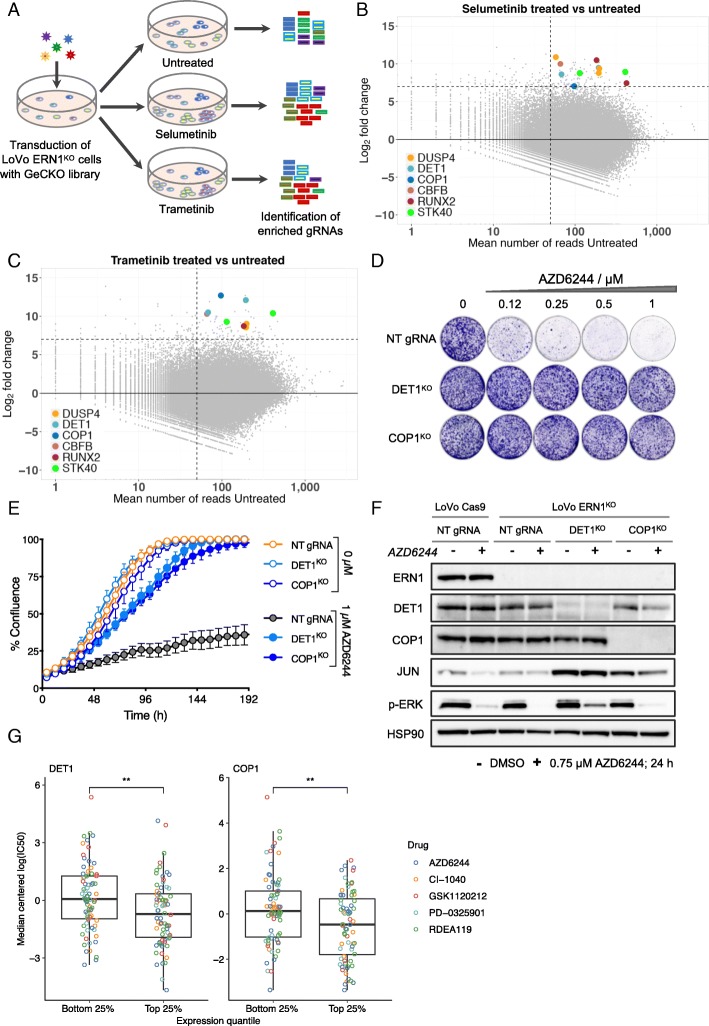
A genetic screen for resistance to MEK inhibitors in ERN1 knockout colon cancer. **a** Schematic outline of the genome-scale CRISPR/Cas9 knockout screen for resistance to MEK inhibition. Two different MEK inhibitors, selumetinib and trametinib, were used, each in two replicates, and compared to the untreated control population. **b**, **c** MA plots of the selumetinib (**b**) and trametinib screens (**c**). Horizontal dashed line indicates an arbitrarily imposed threshold of log_2_ (fold change of treated over untreated) of 7 and vertical dashed line indicates mean number of reads in untreated samples of 50. Highlighted in color are the sgRNAs targeting *DUSP4*, *DET1*, *COP1*, *CBFB*, *RUNX2*, and *STK40*, that are found above these two thresholds (with the *p* adjusted of ≤ 0.1) in both the selumetinib (**b**) and the trametinib (**c**) screen. **d**, **e** Functional validation of DET1 and COP1 in LoVo *ERN1*^KO^ background. **d** Colony formation assays of *DET1* and *COP1* KO cells in the presence and absence of the MEK inhibitor AZD6244 (selumetinib) are shown relative to control cells having NT gRNA. Shown is a representative example of at least three biological replicates. **e** Live cell proliferation assay of *DET1* and *COP1* KO cells in the presence and absence of 1 μM AZD6244 compared to control cells expressing NT gRNA. Error bars indicate standard deviation calculated from three replicate experiments. **f** Western blot analysis of DET1 and COP1 expression in DET1 and COP1 knockout cells using antibodies against ERN1, DET1, COP1, JUN, p-ERK, and HSP90 as control both in the presence and absence of the MEK inhibitor AZD6244. **g** Median-centered log(IC_50_) of five different MEK1 inhibitors in high (top 25%) and low (bottom 25%) expressing DET1 (left) and COP1 (right) CRC cell lines in the GDSC100 data set [42]. Cell lines with high DET1 or COP1 expression have significantly lower IC_50_s (*p* = 0.004 for both DET1 and COP1). Log(IC_50_) estimates were median-centered over all cell lines to make them comparable between MEK inhibitors

To identify a mechanistic link between the ERN1 and the RAF/MEK/ERK signaling pathway, we performed a genome-scale CRISPR/Cas9 MEK inhibitor resistance screen using *ERN1*^KO^ LoVo cells. We screened in the presence and absence of two different MEK inhibitors, selumetinib and trametinib (Fig. 3a) and used differential analysis to identify the genes whose knockout confers resistance to MEK inhibitors. Considering that the CRISPR library used contained only three sgRNAs per gene target, we decided not to impose the criterion of multiple sgRNAs per gene. Nevertheless, we found that four hits (DET1, DUSP4, RUNX2 and STK40) were represented by multiple different sgRNAs, while two hits (COP1 and CBFB) each scored with a single sgRNA both in selumetinib and in trametinib screen (Fig. 3b and c). A complete list of screen results can be found in Additional file 1: Tables S8 and S9.

Dual specificity phosphatase-4 (DUSP4) has been previously implicated in regulating the response to MEK inhibitors, validating the screen performed here [30, 31]. Serine/threonine kinase 40 (STK40) is a negative regulator of NF-κB [32, 33], and NF-κB activity was already shown to directly modulate resistance to several different MAPK pathway inhibitors [34]. In contrast, the remaining four genes (*DET1*, *COP1*, *CBFB*, and *RUNX2*) have not previously been implicated in MAPK signaling or MEK inhibitor resistance. Interestingly, these four genes code for proteins that act pair-wise in complex with each other. The functional and physical interaction between RUNX2 (also known as core-binding factor subunit alpha-1 or CBFA1), and its transcriptional co-activator CBFB (core-binding factor subunit beta) has been well documented using various in vitro [35] and in vivo model systems [36–39]. DET1 and COP1 are part of an E3 ubiquitin ligase complex that promotes ubiquitination and degradation of the proto-oncogenic transcription factor JUN [40]. Because of a previously established link between ERN1 and JNK [41], we studied DET1 and COP1 further to understand the effects of ERN1 loss on the response to MEK inhibitors.

### DET1 and COP1 are regulators of MEK inhibitor response

To validate the results of the genetic screen, we knocked out DET1 and COP1 in *ERN1*-deficient LoVo cells. Importantly, both in long-term assays (Fig. 3d, and Additional file 2: Figure S3) and in short-term assays (Fig. 3e) loss of either DET1 or COP1 conferred resistance to selumetinib and trametinib in these cells. Both vectors were effective in knocking out their respective targets in a polyclonal knockout cell population (Fig. 3f). In addition, biochemical analysis revealed higher basal JUN levels in DET1- and COP1-negative cell populations, consistent with the fact that DET1 and COP1 are part of an E3 ubiquitin ligase complex that degrades JUN [40]. Moreover, computational analyses of drug response data in a large cancer cell line panel [42] further supports that high *DET1* or *COP1* expression is correlated with low IC_50_ values (i.e., sensitivity) for five different MEK inhibitors across a colorectal cancer cell line panel (Fig. 3g).

**Fig. 4:**
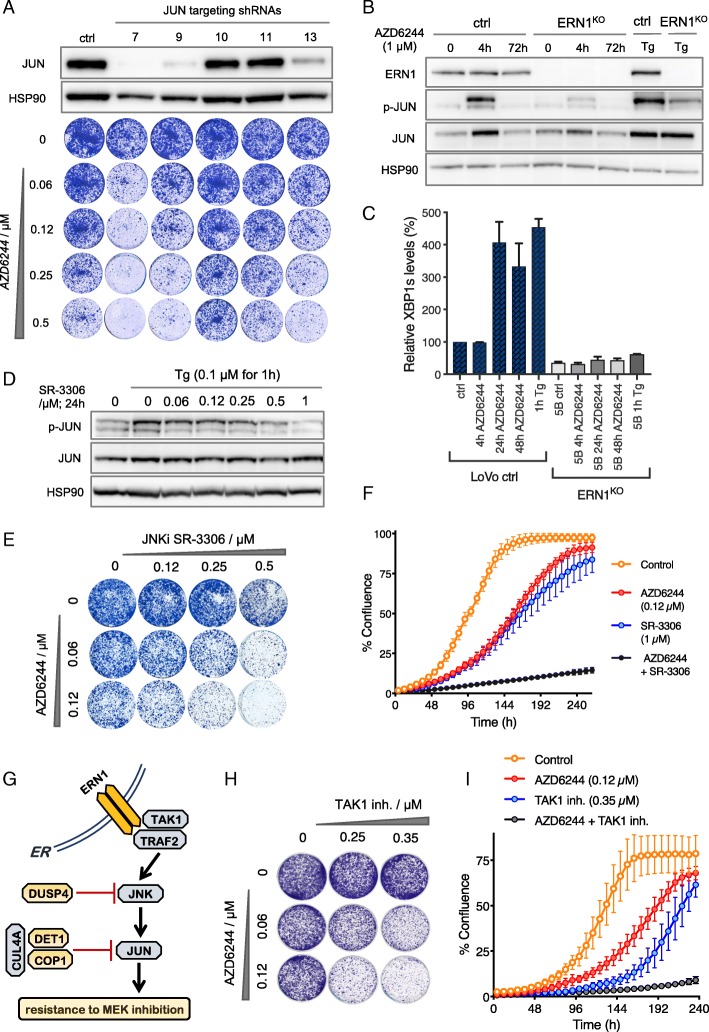
Effect of genetic and pharmacologic downregulation of JUN on response to MEK inhibition. **a** Five different *JUN* targeting shRNAs were used to downregulate *JUN* in LoVo cells. JUN protein levels were quantified by western blotting (top), and the response to increasing concentrations of the MEK inhibitor AZD6244 on *JUN* knockdown cells is shown in colony formation assay (bottom). Empty vector infected control (ctrl) cells are shown here for comparison. **b** Biochemical analysis comparing ERN1^KO^ cells with their control counterparts (ctrl) in the presence and absence of the MEK inhibitor AZD6244 for the indicated number of hours. One-hour thapsigargin treatment (Tg) at 0.1 μM was used as a control for p-JUN induction. **c** Quantification of spliced XBP1 mRNA (XBP1s) in the presence and absence of 1 μM AZD6244 at indicated time points. Error bars indicate standard deviation calculated from three replicate experiments. **d** Biochemical analysis of JUN phosphorylation in the presence and absence of increasing concentrations of the JNK inhibitor SR-3306. One-hour of thapsigargin treatment (Tg) at 0.1 μM was used for p-JUN induction. **e** A representative colony formation assay of LoVo cells grown in the increasing concentrations of the JNK inhibitor SR-3306 (horizontally) and the increasing concentrations of the MEK inhibitor AZD6244 (vertically). **f** Live cell proliferation assay for the combination of the MEK inhibitor AZD6244 and the JNK inhibitor SR-3306 (black), each inhibitor individually (red and blue), and vehicle-treated control cells (yellow line). Error bars indicate standard deviation calculated from three replicate experiments. **g** Schematic representation of the signaling from the endoplasmic reticulum (ER) embedded ERN1 to JNK and JUN via its binding factor TRAF2 and TAK1. Shown in yellow are resistance screen hits DUSP4, DET1, and COP1, which are all negative regulators of JNK and JUN, respectively. **h** A representative colony formation assay showing the effect of the TAK1 inhibitor (5Z)-7-oxozeanol (5ZO) on the proliferation of *KRAS* mutant LoVo cells in the presence of the indicated concentrations of the MEK inhibitor AZD6244. **i** Live cell proliferation assay for the combination of the MEK inhibitor AZD6244 and TAK1 inhibitor 5ZO over the course of 10 days (240 h). Yellow line shows vehicle-treated control cells. Error bars indicate standard deviation calculated from three replicate experiments

Besides an endonuclease and a kinase function, human ERN1 regulates JNK signaling through binding of the adaptor protein TRAF2 [41], which activates JNK to phosphorylate the transcription factor JUN. We tested if active JNK signaling is important for MEK inhibitor sensitivity by directly knocking down *JUN* using shRNAs. We found that LoVo cells are dependent on JUN for proliferation upon treatment with MEK inhibitor. Importantly, the sensitivity of the LoVo cells to treatment with MEK inhibitor correlated with the levels of JUN protein (Fig. 4a). To investigate if ERN1 is required for the activation of JUN, we compared JUN phosphorylation in ERN1^KO^ cells to control cells, in the presence and absence of MEK inhibitor. We observed a strong increase in JUN phosphorylation in ERN1 WT cells, compared to ERN1^KO^ cells, after 4 h of MEK inhibitor treatment (Fig. 4b). Consistently, we found that JUN expression is increased by MEK inhibitor in parental cells, but not in ERN1^KO^ cells, which is not caused by expression changes of either *JNK1* or *JNK2* mRNA (Additional file 2: Figure S4 and S5). These results indicate that ERN1-deficient cells are unable to fully activate JUN signaling, which may explain the MEK inhibitor sensitivity of ERN1^KO^ cells. Moreover, we found that MEK inhibitor treatment induces ERN1 activity, an effect not seen in ERN1^KO^ cells (Fig. 4c).

Finally, we tested if directly inhibiting JNK kinase signaling with a JNK kinase inhibitor would sensitize LoVo cells to MEK inhibition. The potency and specificity of the JNK inhibitor SR-3306 was tested by measuring phosphorylated JUN levels upon treatment of cells with the ER stress-inducing agent thapsigargin (Tg) (Fig. 4d). We found that LoVo cells were sensitive to the combination of JNK and MEK inhibition (Fig. 4e, f). This effect was also found by blocking TAK1, a kinase upstream of JNK (Fig. 4g–i).

## Discussion

Although the yeast and human *RAS* genes have many properties that are interchangeable, the signaling pathways that are controlled by them differ. Here, we find that both yeast and human RAS share a link with the UPR. The shared interaction suggests that an analogous genetic network structure evolved connecting both yeast and human RAS to the ER stress signaling. Using genome-wide synthetic lethality screens in yeast, we identified multiple genes necessary for ER homeostasis, including the UPR stress sensor *IRE1*, to be SL with mutant *RAS*. This genetic interaction was not observed in *KRAS* mutant colon cancer cells, which are unaffected by genetic ablation of *ERN1*, the human ortholog of *IRE1*. However, in contrast to yeast, human cells possess a RAF/MEK/ERK MAPK pathway, and inhibiting this pathway uncovers the SL interaction between *ERN1*^KO^ and mutant *KRAS*. Although we conclude that *ERN1* itself is dispensable for cell growth and proliferation, we find that its loss can sensitize *KRAS* mutant colon cancer cells to MEK inhibition. Considering unsatisfactory performance of MEK inhibitors in clinical trials [43–45], we used *ERN1* knockout colon cancer cells as a model to study resistance mechanisms to MEK inhibition. As small molecule ERN1 inhibitors failed to enhance sensitivity to MEK inhibition, we resorted to genetic screens to explore the mechanism responsible for the observed synthetic lethality effect. Our genome-wide CRISPR/Cas9 screen identified a series of genetic events that can reinstate MEK inhibitor resistance in *ERN1* knockout colon cancer cells. One of the most prominent hits in this screen was dual specificity phosphatase-4 (DUSP4), a well-established tumor suppressor that negatively regulates JUN N-terminal kinase JNK. Upon loss of DUSP4, derepressed JNK activity stimulates JUN-mediated transcription, leading to aberrant MAPK pathway activation [31]. Interestingly, two other screen hits, DET1 and COP1, are also negative regulators of JUN.

Originally described as regulators of light signaling in *Arabidopsis thaliana* [46], both DET1 (de-etiolated homolog 1) and COP1 (constitutive photomorphogenic 1, also known as RFWD2) mechanistically function as E3 ubiquitin-protein ligases and are evolutionarily conserved members of the COP-DET-FUS protein family. Extensive biochemical studies have shown that COP1-DET1 complex targets JUN for ubiquitination and degradation [40]. Further characterization of in vivo models established the role of human *COP1* as a tumor suppressor [47–49]. Here we uncover a role for human COP1 and DET1 in resistance to MEK inhibitors via inhibition of the JNK-JUN pathway.

Since three of the genes identified in our resistance screen (DUSP4, DET1, and COP1) are negative regulators of JUN, we propose that activated ERN1 leads to increased JUN activity, which then translates to cell proliferation despite the inhibition of MEK. ERN1 is linked to the JUN pathway via its binding factor TRAF2, which executes a signaling cascade resulting in the activation of JUN N-terminal kinase JNK [41]. Furthermore, our work demonstrates that the kinase and endonuclease domains of ERN1 are not responsible for the differential sensitivity to MEK inhibition. Recently, we showed that cancers that fail to activate JNK-JUN, due to inactivating mutations in upstream kinases MAP3K1 and MAP2K4, are sensitive to MEK inhibition [50]. Here we demonstrate that *ERN1*^KO^ cells also fail to activate the JNK-JUN pathway resulting in a similar sensitivity to MEK inhibition.

We propose that the JNK arm of MAPK signaling can functionally compensate for the inhibition of the MEK/ERK signaling axis. Conversely, under conditions of abrogated JNK signaling, such as in the presence of JNK or TAK1 inhibitors, cells become more dependent on the flux of signal through the MEK/ERK pathway. This dependency could then prove to be of therapeutic importance. We speculate that cells in which *ERN1* knockout does not sensitize to MEK inhibition (such as DLD1 cells, Additional file 2: Figure S2A-C) can activate JNK-JUN signaling through other pathways, thereby making such cells independent of ERN1 for their MEK inhibitor response. Alternatively, other pathways may be involved in MEK inhibitor resistance in these cells.

We report synergistic cell growth arrest when JNK and MEK inhibitors are combined. Moreover, inhibition of JNK itself (Fig. 4d, e) or JNK activators, such as TAK1 (Fig. 4g, h), might also be useful in preventing intrinsic resistance to MEK inhibitors. In this study, we made use of the resorcyclic lactone (5Z)-7-oxozeanol (5ZO) as a TAK1 inhibitor. However, considerable off-target effects render this molecule inadequate for therapeutic purposes. It remains to be seen whether recently developed TAK1 inhibitors [51] give a more favorable toxicologic profile in the clinic. Taken together, our findings identify an unexpected role for the Unfolded Protein Response executor ERN1 in determining the response to MEK inhibition in *KRAS*-driven colon cancer.

## Conclusions

We identify here a set of genes involved in endosomal transport and ER stress that are synthetic lethal with mutant *RAS* in yeast. At the crossroads of these processes, we identify *IRE1* and *HAC1* that are not only synthetic lethal with hyperactivated RAS signaling in yeast, but also with *ERI1*, a non-essential component of the GPI-GnT enzyme which mediates ER stress response. The fact that *IRE1* and *HAC1* are both master regulators of the unfolded protein response (UPR) indicates that ER homeostasis is disturbed in mutant *RAS* expressing cells and that these cells are dependent on the UPR.

Moreover, in human colon cancer cell lines, we find that MAPK pathway shields *KRAS* mutant cells from synthetic lethality with *ERN1*, a human ortholog of *IRE1*. These interactions point to an evolutionarily conserved genetic network structure between RAS signaling and ER stress.

Finally, we find that ERN1 is an important regulator of JUN activity, which becomes crucial for survival in *KRAS* mutant colon cancer under conditions of abrogated MAPK signaling. We identify the ERN1-JNK-JUN pathway as a novel regulator of MEK inhibitor response in *KRAS* mutant colon cancer, and point to synthetic lethality of MEK inhibition with therapeutics targeting JUN activating kinases, TAK1 and JNK. The genetic network connecting JUN and MAPK signaling may explain why *KRAS* mutant tumor cells are traditionally seen as highly refractory to MEK inhibitor therapy, but these genetic interactions may also provide a therapeutically exploitable vulnerability.

## Additional files

Additional file 1: contains Tables S1-S9. **Table S1.** Results of the primary yeast screen with RAS alleles RAS1 and RAS2 (wild-type) and mutants RAS1(V19) and RAS2(V19). **Table S2.** Results of the validation yeast screen for RAS1. **Table S3.** Results of the validation yeast screen for RAS2. **Table S4.** Results of the validation yeast screen for RAS1(V19). **Table S5.** Results of the validation yeast screen for RAS2(V19). **Table S6.** Gene deletion yeast strains sensitive to ER stress agents (Y. Chen et al., Mol Cancer Res 2005 [23]) and strains sensitive to the expression of RAS2(V19). **Table S7.** Gene deletion yeast strains sensitive to ERI1 deletion (M. Costanzo et al., Science 2010 [25]) and strains sensitive to the expression of RAS2(V19). **Table S8.** Results of the genome-wide CRISPR/Cas9 screen with the MEK inhibitor AZD6244 (selumetinib). **Table S9.** Results of the genome-wide CRISPR/Cas9 screen with the MEK inhibitor trametinib. (XLSX 15504 kb)

Additional file 2: **Figure S1.** Genome-wide synthetic lethal screens with RAS1(V19) and RAS2(V19) identify overlapping sets of genes. **Figure S2.** The response of SW480 ERN1KO and DLD1 ERN1KO KRAS mutant colon cancer cells to MEK inhibition. **Figure S3.** Colony formation assays of *DET1* and *COP1* knockout cells (in LoVo *ERN1*KO background) in the presence and absence of the MEK inhibitor trametinib are shown relative to control cells expressing non-targeting (NT) gRNA. **Figure S4.** Quantification of JUN expression levels in MEK inhibitor (MEKi, 1 μM AZD6244), JNK inhibitor (JNKi, 1 μM SR-3306) and combination treatment (JNKi + MEKi). One-hour thapsigargin treatment (Tg, 100 nM) was used as a control. Error bars represent standard deviation of three replicate experiments. **Figure S5.** Quantification of JNK1 (A) and JNK2 (B) expression levels in MEK inhibitor (MEKi, 1 μM AZD6244), JNK inhibitor (JNKi, 1 μM SR-3306) and combination treatment (JNKi + MEKi). One-hour thapsigargin treatment (Tg, 100 nM) was used as a control. Error bars represent standard deviation of three replicate experiments. (PDF 11800 kb)

## Abbreviations

ER: Endoplasmic reticulum
JNKi: JNK inhibitor
KO: Knockout
MEKi: MEK inhibitor
NT: Non-targeting
PEI: Polyethylenimine
RIDD: Regulated IRE1-dependent decay
SL: Synthetic lethal
SPA: Selective ploidy ablation
Tg: Thapsigargin
UPR: Unfolded protein response
WT: Wild-type
