# Correction to: A role for the unfolded protein response stress sensor ERN1 in regulating the response to MEK inhibitors in KRAS mutant colon cancers

**DOI:** 10.1186/s13073-020-00815-5

**Published:** 2021-02-10

**Authors:** Tonći Šuštić, Sake van Wageningen, Evert Bosdriesz, Robert J. D. Reid, John Dittmar, Cor Lieftink, Roderick L. Beijersbergen, Lodewyk F. A. Wessels, Rodney Rothstein, René Bernards

**Affiliations:** 1 Division of Molecular Carcinogenesis, Oncode Institute, The Netherlands Cancer Institute, Plesmanlaan 121, Amsterdam, 1066 CX The Netherlands; 2 Department Genetics and Development, Columbia University Vagelos College of Physicians & Surgeons, New York, NY 10032 USA

**Correction to: Genome Med (2018) 10: 90**

**https://doi.org/10.1186/s13073-018-0600-z**

It was highlighted that the original article [1] contained an error in Fig. 4a. Specifically, in the experiment shown in Fig. 4a, six concentrations of MEK inhibitor were tested: 0, 30, 60, 125, 250 and 500 uM. Five of these concentrations were shown, but for shRNA10 one dish was accidentally used twice. The 0,25 uM dish has been replaced with the correct one. In the figure they were labeled 0, 0.06, 0.12, 0.25 and 0.5 uM. However, the numbers should be 0, 0.03, 0.06, 0.12 and 0.25. These changes do not affect any of the conclusions of the manuscript. This Correction article shows the correct Fig. 4. The original article has been updated.

**Fig. 4:**
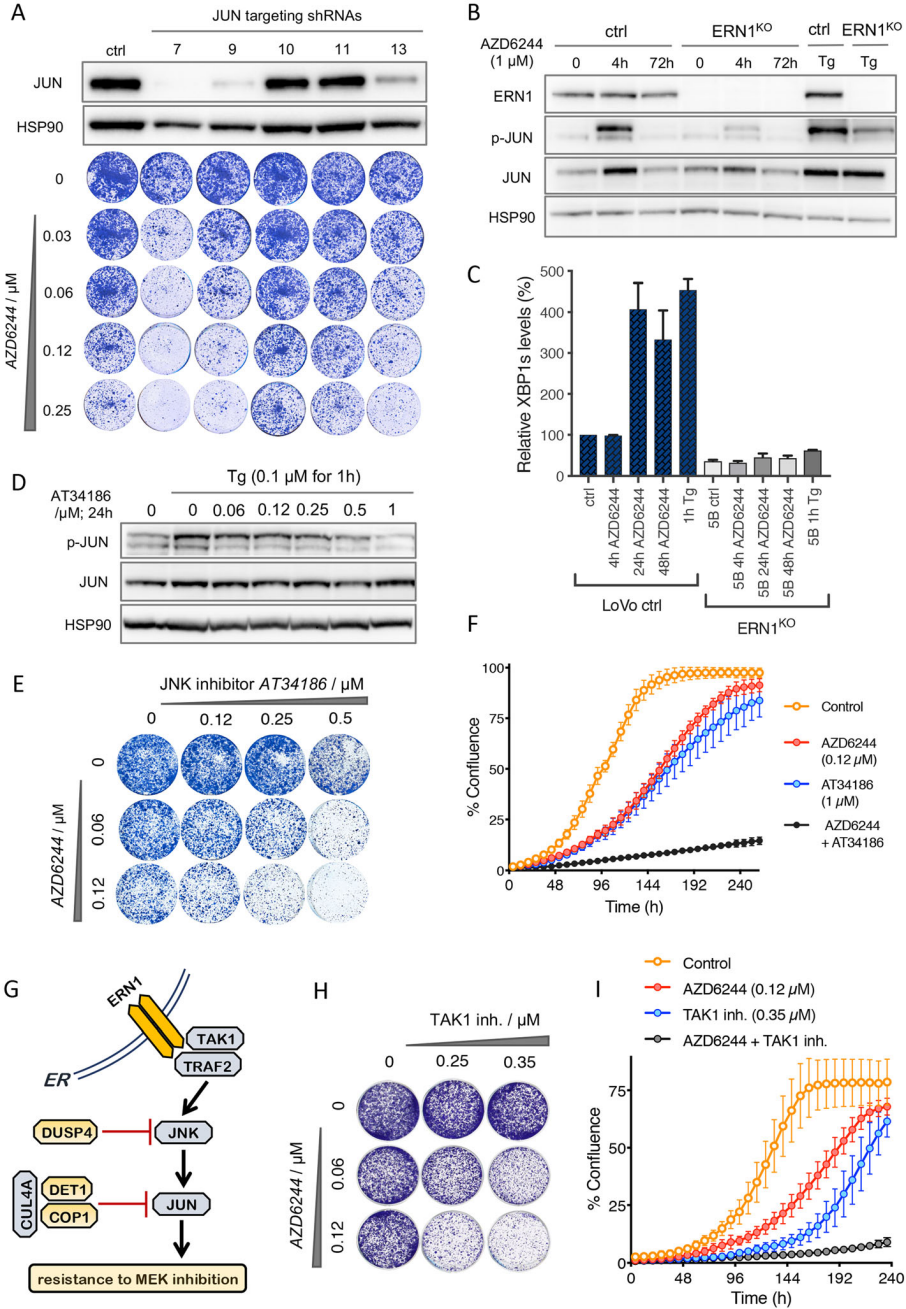
Effect of genetic and pharmacologic downregulation of JUN on response to MEK inhibition. **a** Five different *JUN* targeting shRNAs were used to downregulate *JUN* in LoVo cells. JUN protein levels were quantified by western blotting (top), and the response to increasing concentrations of the MEK inhibitor AZD6244 on *JUN* knockdown cells is shown in colony formation assay (bottom). Empty vector infected control (ctrl) cells are shown here for comparison. **b** Biochemical analysis comparing ERN1^KO^ cells with their control counterparts (ctrl) in the presence and absence of the MEK inhibitor AZD6244 for the indicated number of hours. One-hour thapsigargin treatment (Tg) at 0.1 μM was used as a control for p-JUN induction. **c** Quantification of spliced XBP1 mRNA (XBP1s) in the presence and absence of 1 μM AZD6244 at indicated time points. Error bars indicate standard deviation calculated from three replicate experiments. **d** Biochemical analysis of JUN phosphorylation in the presence and absence of increasing concentrations of the JNK inhibitor SR-3306. One-hour of thapsigargin treatment (Tg) at 0.1 μM was used for p-JUN induction. **e** A representative colony formation assay of LoVo cells grown in the increasing concentrations of the JNK inhibitor SR-3306 (horizontally) and the increasing concentrations of the MEK inhibitor AZD6244 (vertically). **f** Live cell proliferation assay for the combination of the MEK inhibitor AZD6244 and the JNK inhibitor SR-3306 (black), each inhibitor individually (red and blue), and vehicle-treated control cells (yellow line). Error bars indicate standard deviation calculated from three replicate experiments. **g** Schematic representation of the signaling from the endoplasmic reticulum (ER) embedded ERN1 to JNK and JUN via its binding factor TRAF2 and TAK1. Shown in yellow are resistance screen hits DUSP4, DET1, and COP1, which are all negative regulators of JNK and JUN, respectively. **h** A representative colony formation assay showing the effect of the TAK1 inhibitor (5Z)-7-oxozeanol (5ZO) on the proliferation of KRAS mutant LoVo cells in the presence of the indicated concentrations of the MEK inhibitor AZD6244. **i** Live cell proliferation assay for the combination of the MEK inhibitor AZD6244 and TAK1 inhibitor 5ZO over the course of 10 days (240 h). Yellow line shows vehicle-treated control cells. Error bars indicate standard deviation calculated from three replicate experiments

## References

[1] Šuštić T (2018). A role for the unfolded protein response stress sensor ERN1 in regulating the response to MEK inhibitors in KRAS mutant colon cancers. Genome Med.

