# Supplementary material for: A role for the unfolded protein response stress sensor ERN1 in regulating the response to MEK inhibitors in KRAS mutant colon cancers
Source: Genome Med. 2018 Nov 27;10:90. doi: 10.1186/s13073-018-0600-z (PMC6258447; doi:10.1186/s13073-018-0600-z)
Supplement: Supplementary file 2 — Figure S1. Genome-wide synthetic lethal screens with RAS1(V19) and RAS2(V19) identify overlapping sets of genes. Figure S2. The response of SW480 ERN1KO and DLD1 ERN1KO KRAS mutant colon cancer cells to MEK inhibition. Figure S3. Colony formation assays of DET1 and COP1 knockout cells (in LoVo ERN1KO background) in the presence and absence of the MEK inhibitor trametinib are shown relative to control cells expressing non-targeting (NT) gRNA. Figure S4. Quantification of JUN expression levels in MEK inhibitor (MEKi, 1 μM AZD6244), JNK inhibitor (JNKi, 1 μM SR-3306) and combination treatment (JNKi + MEKi). One-hour thapsigargin treatment (Tg, 100 nM) was used as a control. Error bars represent standard deviation of three replicate experiments. Figure S5. Quantification of JNK1 (A) and JNK2 (B) expression levels in MEK inhibitor (MEKi, 1 μM AZD6244), JNK inhibitor (JNKi, 1 μM SR-3306) and combination treatment (JNKi + MEKi). One-hour thapsigargin treatment (Tg, 100 nM) was used as a control. Error bars represent standard deviation of three replicate experiments. (PDF 11800 kb) [file 13073_2018_600_MOESM2_ESM.pdf]

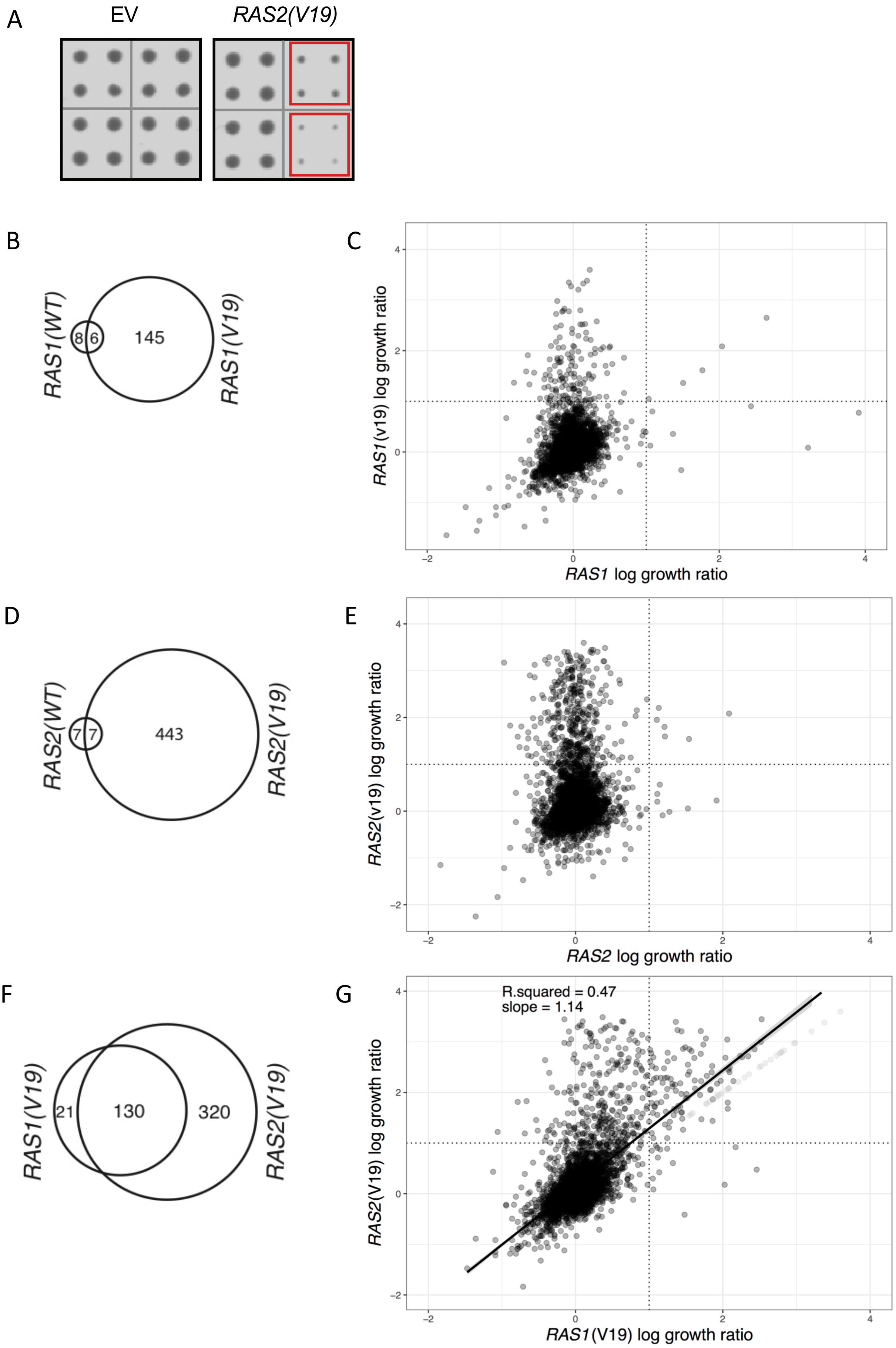

A
EV
RAS2(V19)
B
RAS1(WT)
8
6
145
RAS1(V19)
C
RAS1(v19) log growth ratio
RAS1 log growth ratio
D
RAS2(WT)
7
7
443
RAS2(V19)
E
RAS2(v19) log growth ratio
RAS2 log growth ratio
F
RAS1(V19)
21
130
320
RAS2(V19)
G
R.squared = 0.47
slope = 1.14
RAS2(V19) log growth ratio
RAS1(V19) log growth ratio

Figure S1. Genome-wide synthetic lethal screens with *RAS1(V19)* and *RAS2(V19)* identify overlapping sets of genes.

(A) Yeast strains containing the empty vector (EV) control (left) were used as a growth reference for the same strains expressing *RAS2(V19)* (right). Each of the ~4800 gene deletion strains is represented by 4 colonies. Two strains showing a growth defect in the presence of *RAS2(V19)* are outlined in red.

(B) Venn diagram showing the overlap between an SL screen with *RAS1* wild type (WT), that yielded 14 strains with a growth defect, and mutant *RAS1(V19)* screen that yielded 151 strains with a growth defect.

(C) Plot showing log growth ratios of the *RAS1* and *RAS1(V19)* SL screens. Ratios are calculated as the base 2 log of the growth ratio of the empty vector control divided by *RAS* plasmid, thus higher values represent slower growth when a *RAS* allele is expressed. Dotted lines show the 2-fold growth ratio difference from the population mean.

(D) Overlap between *RAS2* wild type (WT) screen and mutant *RAS2(V19)* screen.

(E) Plot of log growth ratios for the *RAS2* and *RAS2(V19)* SL screens.

(F) Venn diagram showing overlap between two screens with mutant *RAS*, *RAS1(V19)* and *RAS2(V19)*. 130 out of 151 total synthetic lethal interactions from the *RAS1(V19)* screen are also present in the *RAS2(V19)* screen.

(G) Plot of log growth ratios and correlation between the *RAS1(V19)* and *RAS2(V19)* screens. The slow growth phenotype in the population of deletion mutants was overall more severe in the *RAS2(V19)* screen as compared with the *RAS1(V19)* screen. The solid line indicates the calculated correlation (slope and R^2 value listed in inset). There are approximately 30 strains that show no growth with *RAS1*(V19) and *RAS2*(V19) and thus give identical growth ratios, i.e., slope = 1.0 (lighter gray points in the upper right quadrant).

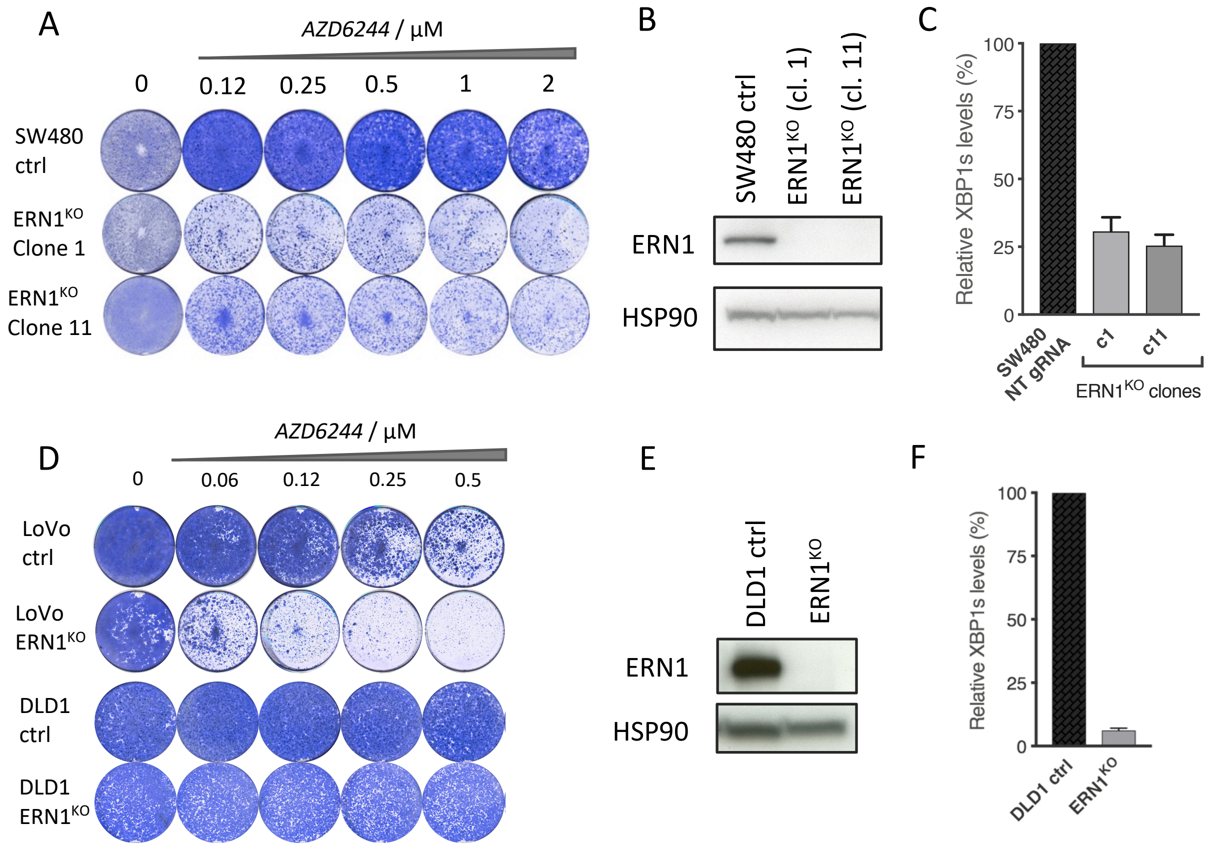

Figure S2. The response of SW480 *ERN1*^{KO} and DLD1 *ERN1*^{KO} *KRAS* mutant colon cancer cells to MEK inhibition.

(A) Colony formation assay of SW480 *ERN1*^{KO} cells in indicated concentrations of the MEK inhibitor AZD6244.

(B) The expression of ERN1 in SW480 *ERN1*^{KO} cells.

(C) Quantification of spliced XBP1 mRNA (XBP1s) in SW480 *ERN1*^{KO} clones.

(D) Colony formation assay of LoVo *ERN1*^{KO} cells and DLD1 *ERN1*^{KO} cells in indicated concentrations of the MEK inhibitor AZD6244.

(E) The expression of ERN1 in DLD1 *ERN1*^{KO} cells.

(F) Quantification of spliced XBP1 mRNA (XBP1s) in DLD1 *ERN1*^{KO} clone.

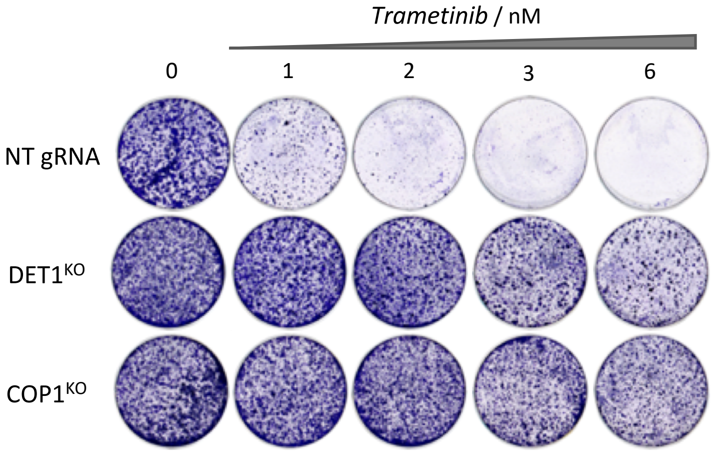

Figure S3. Colony formation assays of *DET1* and *COP1* knockout cells (in LoVo *ERN1*^{KO} background) in the presence and absence of the MEK inhibitor trametinib are shown relative to control cells expressing non-targeting (NT) gRNA.

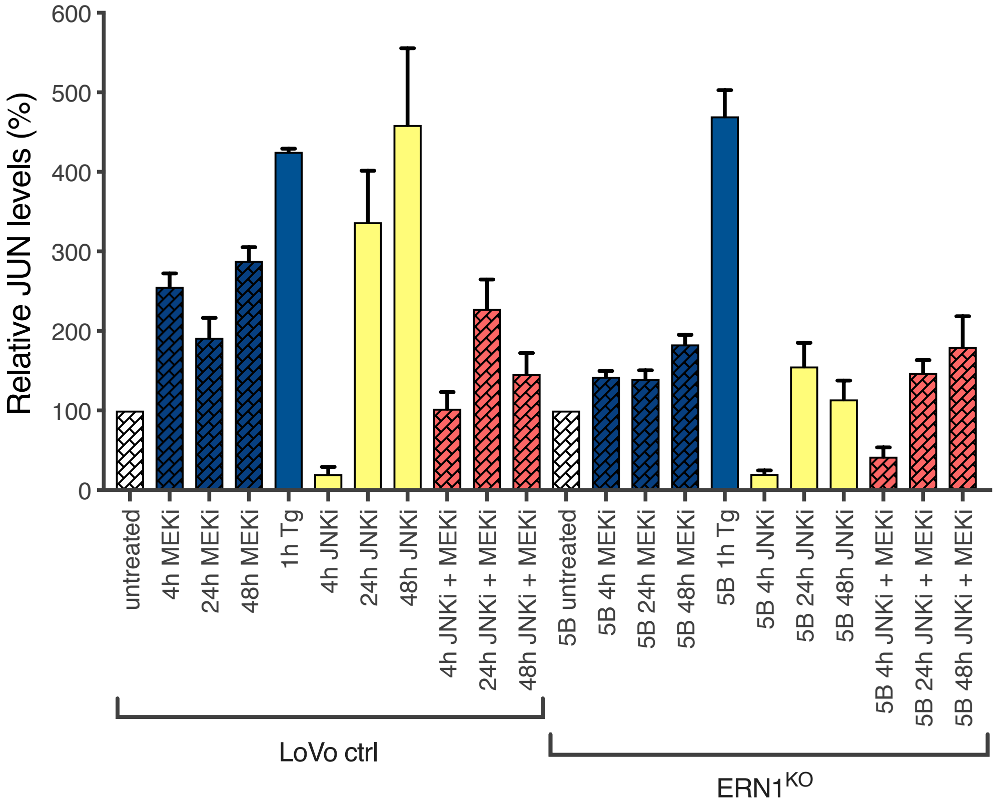

Figure S4. Quantification of JUN expression levels in MEK inhibitor (MEKi, 1 µM AZD6244), JNK inhibitor (JNKi, 1 µM SR-3306) and combination treatment (JNKi + MEKi). One hour thapsigargin treatment (Tg, 100 nM) was used as a control. Error bars represent standard deviation of three replicate experiments.

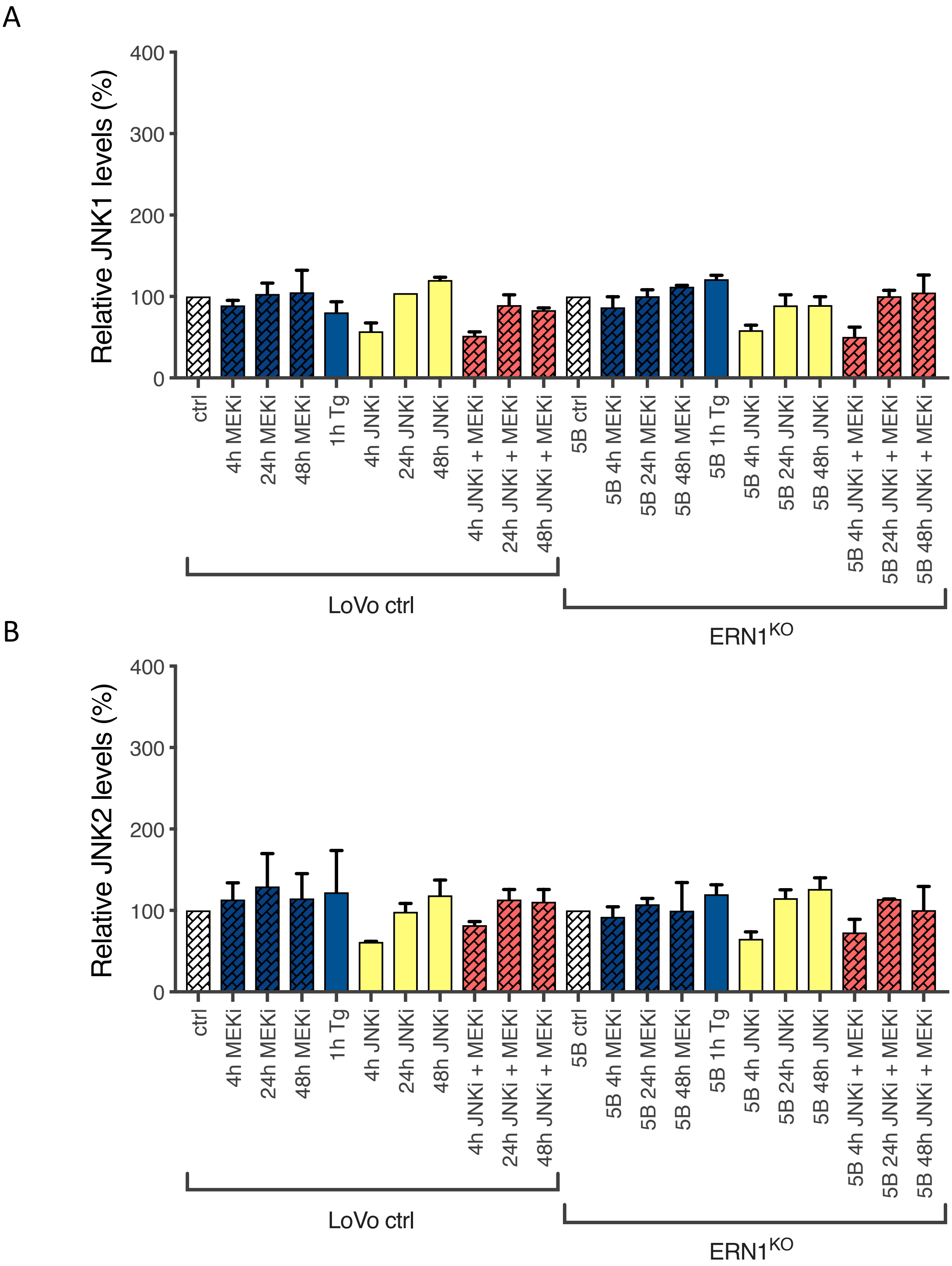

Figure S5. Quantification of JNK1 (A) and JNK2 (B) expression levels in MEK inhibitor (MEKi, 1 µM AZD6244), JNK inhibitor (JNKi, 1 µM SR-3306) and combination treatment (JNKi + MEKi). One hour thapsigargin treatment (Tg, 100 nM) was used as a control. Error bars represent standard deviation of three replicate experiments.
